# Ultrasensitive Dopamine Detection in Undiluted Serum with a Disposable Electrochemical Sensor Employing MOF-Derived Gold Nanocomposites

**DOI:** 10.3390/bios16050255

**Published:** 2026-04-30

**Authors:** Rohan Sagar, Hsiao-Wei Wen, Ching-Chou Wu, M. S. Gaur

**Affiliations:** 1 Department of Bio-Industrial Mechatronics Engineering, National Chung Hsing University, Taichung City 402, Taiwan; rohanphysics@dragon.nchu.edu.tw; 2 Department of Food Science and Biotechnology, National Chung Hsing University, Taichung City 402, Taiwan; hwwen@nchu.edu.tw; 3 Food and Animal Product Safety Inspection Center, National Chung Hsing University, Taichung City 402, Taiwan; 4 Development Center of Sustainable Agriculture, National Chung Hsing University, Taichung City 402, Taiwan; 5 Department of Physics, Hindustan College of Science and Technology, Farah, Mathura 281122, Uttar Pradesh, India; ms.gaur.hcst@sgei.org

**Keywords:** MOF-derived gold nanocomposite, dopamine, electrochemical sensors, screen-printed carbon electrode, undiluted serum

## Abstract

Dopamine (DA) is essential for motor control, motivation, and cognition, and its dysregulation is associated with neurological and psychiatric disorders such as Parkinson’s disease, schizophrenia, and addiction. Accurate and selective DA quantification in complex biological matrices is important, but remains challenging because of coexisting interferents and the low physiological concentration of DA. Here, we report a disposable electrochemical DA sensor based on screen-printed carbon electrodes (SPCEs) modified with metal–organic framework-derived gold nanocomposites (MOFD-AuNCs). The optimal material, synthesized with a 60 min NaBH_4_ reduction step (MOFD-AuNC-60), exhibited superior electron-transfer kinetics compared with materials prepared at other reduction times. A single coating of MOFD-AuNC-60 on SPCEs enabled DA oxidation at a low potential (~0.05 V) with high selectivity in the presence of ascorbic acid and uric acid. In undiluted porcine serum, the sensor exhibited a dynamic range of 2.5–500 nM with a calculated detection limit of 0.5 nM. In undiluted human serum, it exhibited a dynamic range of 5–100 nM with a calculated detection limit of 4.4 nM. The MOFD-AuNC-60/SPCEs further demonstrated excellent reproducibility (relative standard deviation, 3%) and stability (7.5% current loss over 7 days). These results demonstrate that the proposed sensor provides a disposable, robust, and reliable sensing platform for direct DA detection in undiluted serum, showing promise for practical applications.

## 1. Introduction

Dopamine (DA), a neurotransmitter, plays a crucial role in the renal, cardiovascular, and central nervous systems of mammals, influencing various physiological processes [1,2]. For example, high DA levels indicate cardiotoxicity, leading to rapid heart rates, hypertension, and heart failure [3]. In contrast, low levels of DA in the central nervous system are implicated in neurological diseases such as Parkinson’s disease, schizophrenia, Alzheimer’s disease, stress, and depression [4,5]. Detecting DA is essential for determining neurological disorders and brain diseases [6,7]. DA concentrations in human blood and serum are incredibly low, from several nanomolar to sub-micromolar, alongside other electroactive biomolecules, such as ascorbic acid (AA) and uric acid (UA) [8,9]. Therefore, developing ultrasensitive, selective, and convenient DA sensors for whole blood or undiluted serum samples is essential for practical diagnostic applications.

Several analytical methods, including capillary electrophoresis, fluorescence spectroscopy, and liquid chromatography, have been widely used to detect DA, AA, and UA in biological samples [10]. Although these techniques provide excellent selectivity and accuracy, their practical use is often limited by complicated sample pretreatment, the requirement for trained personnel, expensive instrumentation, and long analysis times. In contrast, electrochemical sensing has emerged as a powerful alternative for DA detection due to its simplicity, high sensitivity, rapid response, and compatibility with miniaturized and disposable electrodes [11]. However, in biological matrices, the coexistence of electroactive species, such as AA and UA, can produce overlapping oxidation signals in electrochemical sensing. To improve sensing performance, numerous nanomaterials or nanocomposites (NCs), such as carbon nanocoils (CNCs) and copper tetra(p-methoxyphenyl)porphyrin (CuTMePP) [12], N-TiO_2_@Ag-nanoparticles (NPs)/graphene quantum dot (GQD) [13], AuNPs@MoSe_2_ [14], ZnO NPs, AuNPs@multiwall carbon nanotube (MWCNT)/graphene oxide (GO) [15], and metal–organic framework (MOF)-based NCs [16,17], have been integrated with conventional electrodes to enhance electron transfer and signal resolution. Despite these advances, few studies have achieved nanomolar-level limits of detection (LOD) and, more importantly, demonstrated direct DA detection in undiluted serum, where severe electrode fouling and interference from coexisting electroactive species remain significant challenges.

Currently, much attention has been devoted to MOF NCs due to their wide applications. Specifically, MOFs, consisting of soft organic linkers and metal centers, are advantageous for biosensing due to their porosity, structural tunability, and stability [17,18]. The large surface area and abundant electroactive sites of MOF NCs can enrich analytes and increase electron-transfer rates, which promises the increasing adoption of MOFs in electrochemical sensing applications. Several MOF-based NCs have been employed for DA detection [16,19,20,21,22,23]. Zhou et al. developed GO-AuNPs@Cu-MOF-modified screen-printed carbon electrodes (SPCEs) to achieve an LOD of 16 nM for DA in phosphate-buffered solution (PBS) [16]. Ahmad et al. coated zeolitic imidazolate framework-8 (ZIF-8) onto SPCEs, achieving an LOD of 87 nM for DA and good stability over 30 days [19]. However, they did not disclose the practical applications in serum samples. Wu et al. coated a Cu_3_(HHTP)_2_ MOF, consisting of Cu(II) centers and a redox-active linker (2,3,6,7,10,11-hexahydroxytriphenylene, HHTP), onto a glassy carbon electrode (GCE) for DA detection, achieving an LOD of 5.1 nM in PBS [20]. Ma et al. modified Co/Zn-porphyrin (TCPP) MOF on a GCE to detect DA in PBS with an LOD of 1.67 nM [21]. Furthermore, Hira et al. synthesized MOF-derived (MOFD) Co-N-C NCs for sensitive DA detection using square wave voltammetry (SWV), achieving an LOD of 0.43 μM [22]. Xu et al. grew a leaf-like Co-MOF on carbon cloth (L-Co-MOF/CC) as an electrode, achieving an ultrasensitive LOD of 2.5 nM and demonstrating good recovery at 5 μM DA in 100-fold-diluted human serum [24]. In summary, MOF-based NCs can significantly improve the electrochemical response of DA oxidation, but few DA sensors can directly detect DA in undiluted serum with little interference.

This study proposes MOFD gold NCs, consisting of gold clusters and negatively charged 1,3,5-benzene tricarboxylic acid (BTC) organic linkers, for the selective and sensitive detection of DA in undiluted serum samples. The MOFD-AuNCs were drip-coated onto oxidized SPCEs to construct a disposable DA-sensing strip. The material properties of MOFD-AuNCs were analyzed by Fourier-transform infrared spectroscopy (FTIR), X-ray diffractometry (XRD), transmission electron microscopy (TEM), scanning electron microscopy (SEM), X-ray photoelectron spectroscopy (XPS), and laser diffraction. The sensing properties of MOFD-AuNC-modified SPCEs were evaluated electrochemically using cyclic voltammetry (CV), differential pulse voltammetry (DPV), and electrochemical impedance spectroscopy (EIS). The sensing properties of the MOFD-AuNCs/SPCEs for DA are explored in serum samples.

## 2. Materials and Methods

### 2.1. Materials

Gold (III) chloride trihydrate (HAuCl_4_·3H_2_O), BTC, sodium borohydride (NaBH_4_), DA (>99%), AA (>99%), UA (>99%), ethanol (95%), potassium chloride (KCl), sodium phosphate dibasic (Na_2_HPO_4_), sodium phosphate monobasic dehydrate (NaH_2_PO_4_), potassium hexacyanoferrate(III) (K_3_[Fe(CN)_6_]), hexacyanoferrate(II) trihydrate (K_4_[Fe(CN)_6_]·3H_2_O) were purchased from Sigma-Aldrich (St. Louis, MO, USA). Porcine serum (Catalog No. 26250084, Gibco™, Grand Island, NY, USA) and human serum (AB serotype, Catalog No. 4400056545, Invitrogen, Carlsbad, CA, USA) were bought from Thermo Fisher Scientific (Waltham, MA, USA). Phosphate-buffered solution (PBS, pH 7.0), prepared by 10 mM Na_2_HPO_4_ and 10 mM NaH_2_PO_4_, was used as the background solution for preparing chemicals and electrochemical experiments. All chemicals were reagent-grade and used without additional purification. All solutions were prepared using deionized (DI) water purified with a Milli-Q system, yielding a resistivity of 18.2 MΩ · cm.

### 2.2. MOFD-AuNC Synthesis

**Scheme 1:**
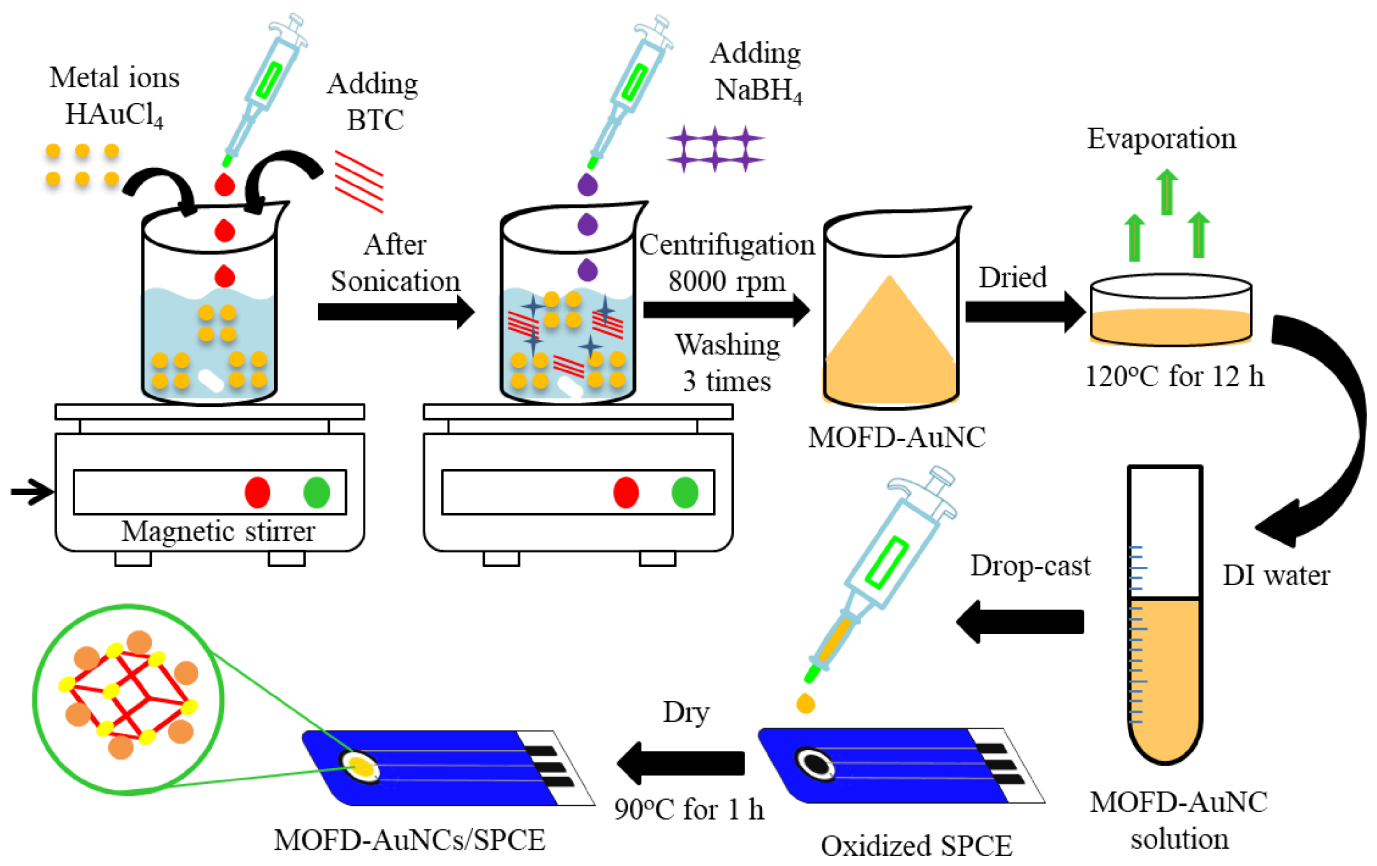
Scheme of preparing MOFD-AuNC and MOFD-AuNC/SPCE.

Following the synthesis of Cu-BTC MOF [25], MOFD-AuNCs were synthesized via a two-step solvent reaction method with minor modifications (Scheme 1). Briefly, 10 mM HAuCl_4_ and 10 mM BTC were separately dissolved in a 25 mL ethanol/DI water mixture (1:1, *v*/*v*), mixed, and stirred at 5000 rpm at 30 °C for 2 h to obtain the Au^3+^-BTC framework. Owing to the high standard reduction potential (+1.5 V versus NHE) of Au^3+^, the Au^3+^-BTC framework is thermodynamically prone to reduction, which could lead to partial Au^0^ formation and framework destabilization. To regulate this reduction process, NaBH_4_ was employed to rapidly reduce the outer Au^3+^ species of the Au^3+^-BTC framework, thereby forming Au-BTC MOFD-AuNCs. An NaBH_4_ solution (3.8 mg/mL) was added dropwise and allowed to react for 30, 60, and 120 min. The resulting products were denoted as MOFD-AuNC-30, MOFD-AuNC-60, and MOFD-AuNC-120, respectively.

The supernatant was removed by centrifugation at 8000 rpm for 300 s, followed by resuspension of the precipitate in DI water. This washing procedure was repeated three times. The collected MOFD-AuNCs were subsequently dried in an oven at 120 °C for 12 h. The resulting MOFD-AuNC powder was stored in airtight containers at 25 °C before use. A 0.02% (*w*/*v*) MOFD-AuNC dispersion was prepared in DI water and sonicated at approximately 20 kHz for 60 min. The prepared dispersion was then stored at 4 °C until further use.

### 2.3. Material Characterization

The morphology of the MOFD-AuNCs was characterized by TEM (JEOL JEM-2010, Tokyo, Japan) and field-emission SEM (JEOL JSM-7401F, Tokyo, Japan) with an accelerating voltage of 3 kV. XPS (PHI 5000 Versa Probe, ULVAC-PHI Inc., Chigasaki, Japan) equipped with an Al Kα X-ray source (excitation energy: 1486.6 eV) was employed to analyze the chemical composition and valence states of the materials. The crystalline faces of the MOFD-AuNCs were analyzed using an in-plane grazing incidence high-resolution XRD (D8 Discover SSS, Bruker AXS, Karlsruhe, Germany) with a 1° resolution using Cu Kα radiation (λ = 1.5418 Å). FTIR spectroscopy (PerkinElmer Spectrum 100, Waltham, MA, USA) was employed to verify the chemical functional groups. The size distribution of the MOFD-AuNCs in DI water was determined using a dynamic light scattering analyzer (Zetasizer, Malvern Panalytical Co., Westborough, MA, USA). A 2 mL aliquot of the MOFD-AuNC solution was placed in a plastic cuvette and analyzed at 25 °C.

### 2.4. Fabrication of MOFD-AuNC-Modified SPCE

SPCEs are ideal candidates for developing disposable sensing strips due to their ease of mass production, cost-effectiveness, and compatibility with portable electrochemical readers. The oxidation procedure for SPCEs, as reported in our previous studies, increases the hydrophilicity and electron-transfer rate of SPCE surfaces [26]. The working electrode (WE) of a three-electrode SPCE (TE100, Zensor R&D, Taichung, Taiwan) was activated by a cyclic potential from 0 to 1.3 V for 20 cycles with a 0.1 V/s scanning rate and then oxidized at 2.0 V for 300 s in 0.1 M PBS (pH 7.0) with a three-electrode system using a Pt plate as the counter electrode (CE) and a commercial Ag/AgCl reference electrode (RE). Subsequently, the oxidized WEs were dripped with the 20 μL aliquot of MOFD-AuNC solution, as shown in Scheme 1. Then, the SPCEs were dried in an oven at 90 °C for 60 min.

### 2.5. Electrochemical Measurement

Electrochemical measurements were performed using a multi-channel potentiostat (Multi Palm Sens4, Palm Sens BV, Houten, The Netherlands). The commercial SPCEs, consisting of a 3 mm diameter WE, a carbon CE, and a silver pseudo-RE, were used for electrochemical DA detection. A homemade 5 mm thick hollow poly(methyl acrylate) plate was attached to the electrode region of the SPCEs, forming a 5 mm-diameter container. A 60 μL aliquot of the DA-containing samples was dripped into the container, enabling rapid electrochemical measurements. CV and DPV measurements were conducted within a potential range of −0.1 V to +0.6 V at a scan rate of 50 mV/s. EIS was performed over a frequency range of 0.1 Hz to 100 kHz with an amplitude of 5 mV in PBS containing 2.5 mM K_3_[Fe(CN)_6_]/K_4_[Fe(CN)_6_].

## 3. Results and Discussion

### 3.1. Material Properties of MOFD-AuNCs

MOF, composed of metal nodes coordinated with negatively charged organic ligands, can form crystalline structures with well-defined geometries. Figure S1 (Supplementary Information) presents TEM images of the Au^3+^-BTC NCs and MOFD-AuNC-60. The Au^3+^-BTC nanocrystals exhibit irregular morphology and a broad size distribution, indicating limited structural stability. In contrast, MOFD-AuNC-60 displays well-defined octahedral-like structures with clear lattice fringes and characteristic interplanar spacings, confirming the presence of Au nanoclusters embedded within the MOFD framework. The Au nanoclusters are uniformly dispersed without obvious aggregation, suggesting effective coordination and stabilization by the BTC linkers, which contribute to the formation of a structurally stable MOFD-AuNC architecture.

Figure S2 (Supplementary Information) shows the FTIR spectra of Au^3+^-BTC NCs and MOFD-AuNC-60, both of which exhibit similar characteristic absorption bands. The peak at approximately 1690 cm^−1^ is assigned to the C=O stretching vibration of residual carboxylic acid groups, while the broad absorption band observed in the range of 2500–3200 cm^−1^ is attributed to O–H stretching vibration [27]. In addition, the band at around 1400–1450 cm^−1^ can be assigned to the symmetric stretching vibration of COO^−^ groups, indicating the participation of carboxylate groups in coordination with Au species [28]. The absorption near 1610 cm^−1^ is associated with aromatic C=C stretching and may also partially overlap with the asymmetric stretching vibration of coordinated carboxylate groups. Moreover, the peaks in the range of 700–800 cm^−1^ are attributed to aromatic C–H out-of-plane bending vibrations, suggesting that the aromatic backbone of BTC was retained. Overall, these FTIR results suggest that both Au^3+^-BTC NCs and MOFD-AuNC-60 possess similar BTC-based structural features and coordination environments.

**Figure 1:**
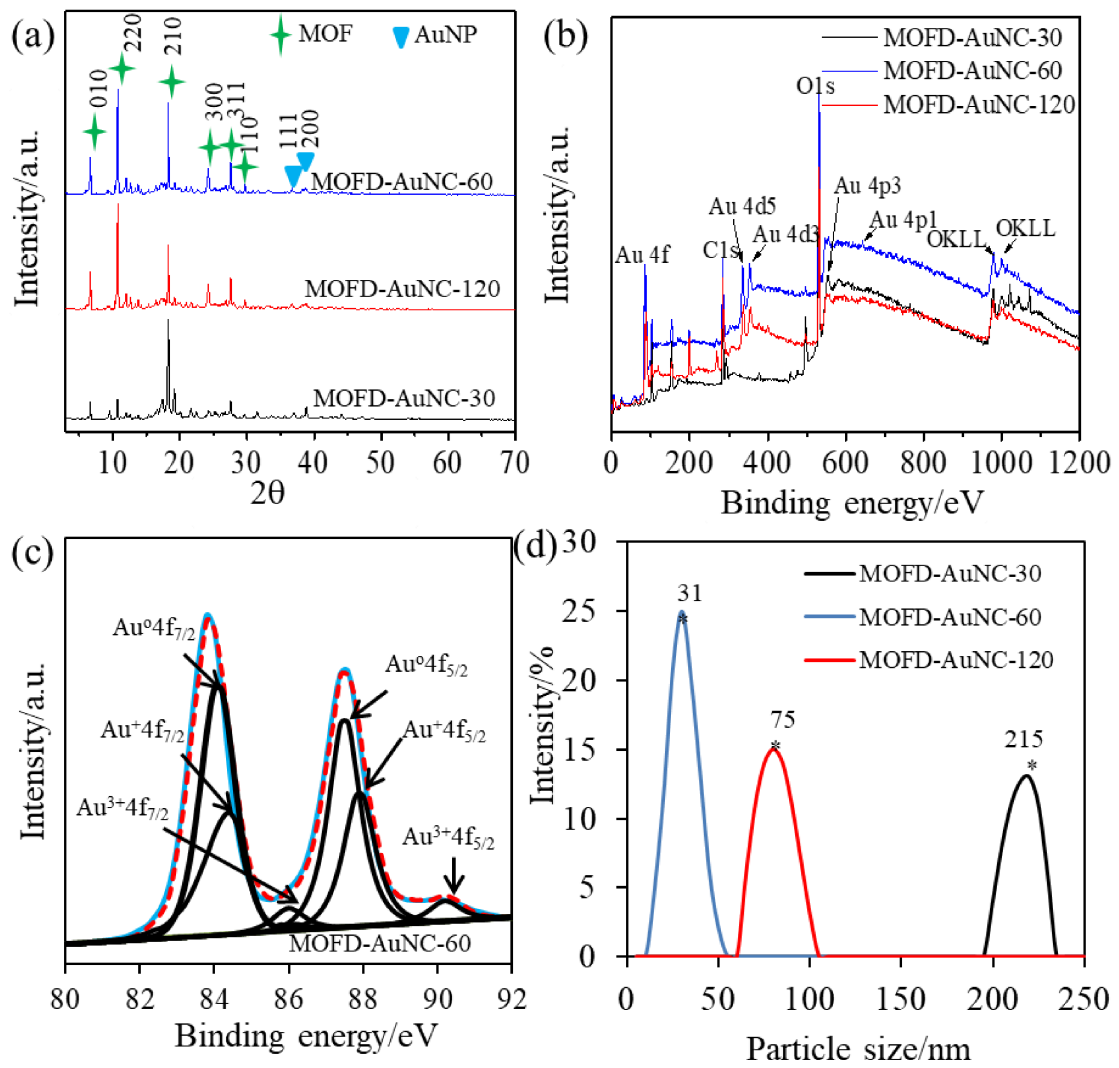
(**a**) XRD spectra and (**b**) XPS spectra of MOFD-AuNC-30 (black), MOFD-AuNC-60 (red), and MOFD-AuNC-120 (green). (**c**) High-resolution XPS spectra of the Au 4f peak of the MOFD-AuNC-60. Blue line: raw data, red line: sum of fitting peaks, black line: fitting peaks. (**d**) Hydrodynamic size analysis of MOFD-AuNCs. The asterisk (*) denotes the peak size value.

Figure 1a shows the XRD patterns of MOFD-AuNCs prepared under different reduction durations. The characteristic 2θ reflections corresponding to crystalline BTC, MOF frameworks, and AuNPs were compared with those from previous studies and summarized in Table S1 (Supplementary Information) [29,30,31,32]. The diffraction peaks indexed as (010), (220), (210), (300), (311), (110), and (111) are consistent with those reported for BTC-based MOF structures in related MOFD-NCs [29,30,31,32], indicating the preservation of MOF crystallographic features in all MOFD-AuNC samples. As summarized in Table S1, the relative crystallinity of MOFD-AuNC-60 and MOFD-AuNC-120 is higher than that of MOFD-AuNC-30, suggesting that reduction duration of at least 60 min favors the development of more ordered MOF periodic structures. In addition, a weak diffraction peak at 2θ of ~38.5–38.8°, corresponding to the Au (111) plane, is attributed to the ultrasmall size and high dispersion of Au nanoclusters within the MOFD framework.

Figure 1b shows XPS spectra of various MOFD-AuNCs, specifically highlighting Au, O, and C as the primary components. Notably, the Au 4f and Au 4d intensity of the binding energy (BE) spectrum obtained from MOFD-AuNC-60 and MOFD-AuNC-120 is much larger than that of MOFD-AuNC-30, suggesting that the 60 min reduction forms significant gold core centers of MOFD-AuNCs. Furthermore, Figure 1c shows the high-resolution XPS spectra of the Au 4f peak of the MOFD-AuNC-60. The fitting peaks at 84.2 and 87.6 eV are attributed to elemental gold (Au^0^). The other two pairs correspond to the oxidation states of gold Au^+^ (BEs at 84.6 and 88 eV) [31] and Au^3+^ (BEs at 86 and 90.4 eV) [33]. Based on the relative peak areas, the atomic percentages are 88.6% for Au^0^, 6.8% for Au^+^, and 4.6% for Au^3+^. The results indicate that the gold core of the MOF has a different valence state. The Au^+^ and Au^3+^ oxidation states of the MOF can exhibit catalytic activity for alkyne carboxylation and propargylamine synthesis, respectively [34,35]. To the best of our knowledge, few studies have reported the effect of Au^+^ and Au^3+^ on dopamine oxidation.

The hydrodynamic size of MOFD-AuNCs dispersed in DI water was evaluated by dynamic light scattering after 1 h of sonication. The corresponding size distribution profiles are shown in Figure 1d. The average diameters of MOFD-AuNC-30, MOFD-AuNC-60, and MOFD-AuNC-120 were determined to be approximately 215 nm, 31 nm, and 75 nm, respectively. The dispersion size of MOFD-AuNCs is influenced by the coordination environment between Au species and BTC ligands, as well as electrostatic and polar interactions within the NC framework. Notably, MOFD-AuNC-60 exhibits a narrower size distribution compared with MOFD-AuNC-30 and MOFD-AuNC-120, indicating improved size uniformity. Owing to its relatively small hydrodynamic size, narrow size distribution, and favorable dispersion stability, MOFD-AuNC-60 was selected for subsequent modification of the SPCE.

### 3.2. Morphology of MOFD-AuNC/SPCEs

The SEM images of the bare oxidized SPCE and the MOFD-AuNC-60-coated SPCE are shown in Figure S3 (Supplementary Information). The bare SPCE surface features micrometer-scale graphite flakes interspersed with nanoscale carbon black particles (Figure S3a), resulting in a relatively flat but heterogeneous morphology. After coating the MOFD-AuNC-60, coral-like porous nanostructures uniformly occupied the SPCE surface, as shown in Figure S3b. The porous architecture increases the electroactive surface area of the modified electrode, thereby improving biosensor sensitivity.

### 3.3. Electrochemical Properties of MOFD-AuNC/SPCEs

**Figure 2:**
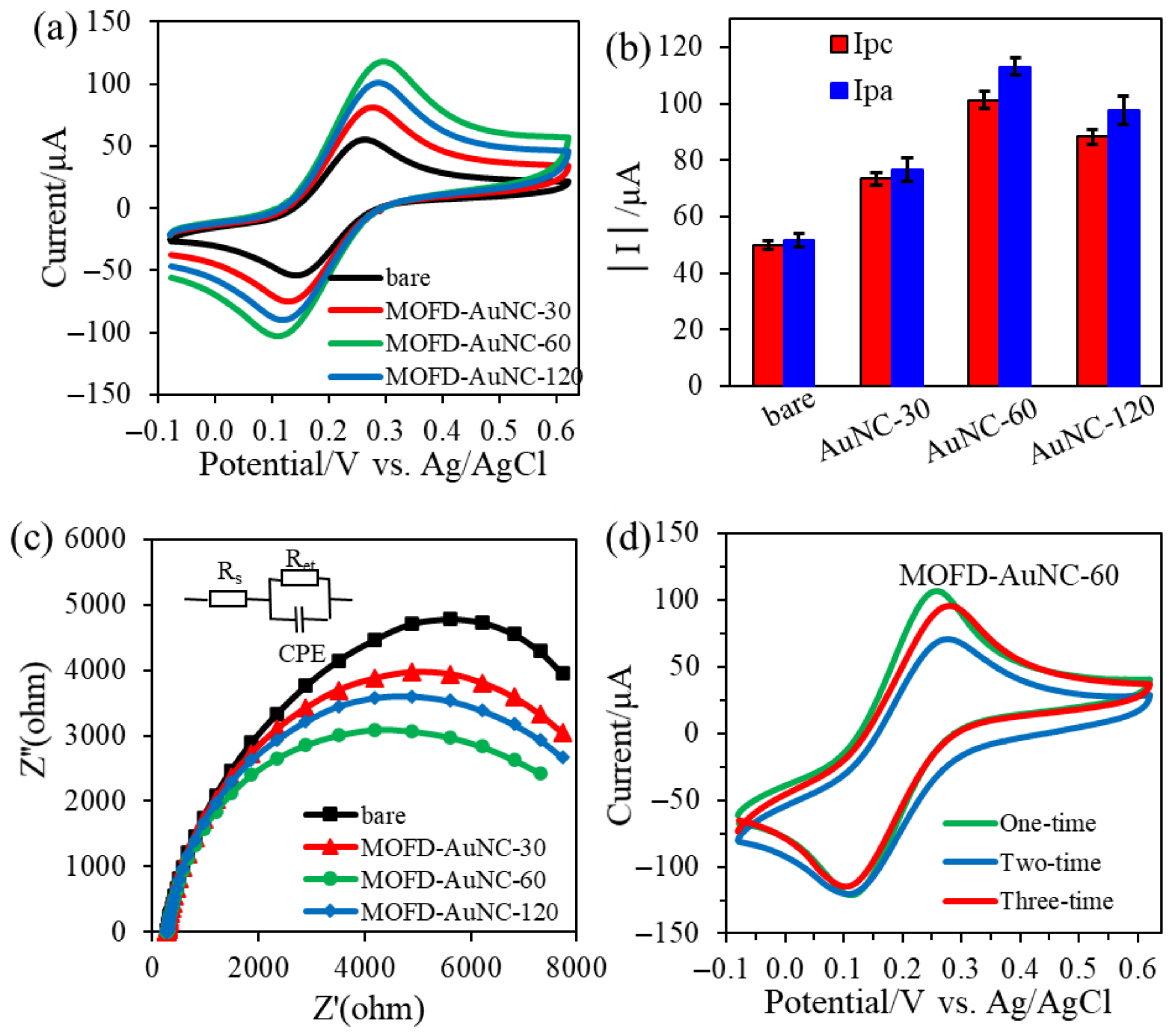
Cyclic voltammograms (**a**), the corresponding anodic (blue) and cathodic peak (red) currents (**b**), and EIS Nyquist plots (**c**) obtained at the bare (black), MOFD-AuNC-30 (red), MOFD-AuNC-60 (green), and MOFD-AuNC-120 (blue)-coated SPCEs (*n* = 3). The inset shows the 1R//C equivalent circuit. (**d**) Cyclic voltammograms measured at the SPCEs with drip-coating of the MOFD-AuNC-60 solution for 1, 2, and 3 times. All measurement was in 2.5 mM K_3_[Fe(CN)_6_]/K_4_[Fe(CN)_6_]-containing PBS. The scanning rate is 50 mV/s.

To investigate the electrochemical properties of MOFD-AuNCs, CV and EIS were performed on bare, MOFD-AuNC-30-, MOFD-AuNC-60-, and MOFD-AuNC-120-coated SPCEs, as shown in Figure 2a,c. The MOFD-AuNC-60/SPCEs show the largest redox peak current with the anodic peak current (I_pa_) of 112.9 ± 2.1 μA (*n* = 3) and the cathodic peak current (I_pc_) of 100.2 ± 2.8 μA (*n* = 3), compared to the bare, the MOFD-AuNC-30, and the MOFD-AuNC-120/SPCEs, as statistically analyzed in Figure 2b. The results suggest that the MOFD-AuNC-60 coating increases the WE surface area more effectively than the MOFD-AuNC-30 and MOFD-AuNC-120 coatings. Moreover, Nyquist plots from EIS measurements show a consistent trend. The MOFD-AuNC-60 coating exhibits a semicircle smaller than those of other coatings, indicating a lower electron-transfer resistance (R_et_) [26]. Notably, no diffusion-controlled linear region was observed in the low-frequency range of the Nyquist plots, which may be associated with electrostatic repulsion between the negatively charged BTC residues on the MOFD-AuNC surface and the [Fe(CN)_6_]^3−^/[Fe(CN)_6_]^4−^ redox couple, leading to increased interfacial impedance. Therefore, the semicircle portion of the impedance spectra was fitted using a simplified Randles-type equivalent circuit, called a 1R//C circuit, composed of a solution resistor (R_s_) in series with a parallel combination of R_et_ and constant phase element (CPE) [26], as shown in the inset of Figure 2c. The R_et_ value obtained from the MOFD-AuNC-60/SPCE is 4.0 ± 0.02 kΩ smaller than that of the bare SPCE (6.5 ± 0.01 kΩ), the MOFD-AuNC-30/SPCE (5.4 ± 0.04 kΩ), and the MOFD-AuNC-120/SPCE (4.9 ± 0.06 kΩ). This result indicates that the MOFD-AuNC-60 coating can significantly reduce the R_et_ value due to its high conductivity and large roughness. According to the above findings, the MOFD-AuNC-60/SPCEs were selected as the preferred electrode for DA detection.

Furthermore, Figure 2d shows the effect of the MOFD-AuNC-60 coating times on the electrochemical response of electrodes. The results indicate that the single-coating MOFD-AuNC-60/SPCE exhibits a smaller redox peak-potential separation (E_pa_–E_pc_) and a larger I_pa_ than the double and triple-coating MOFD-AuNC-60/SPCEs, implying a smaller overpotential and a faster electron-transfer rate. Therefore, the one-time coating of the MOFD-AuNC-60 is used to prepare DA sensors.

### 3.4. DA Detection

**Figure 3:**
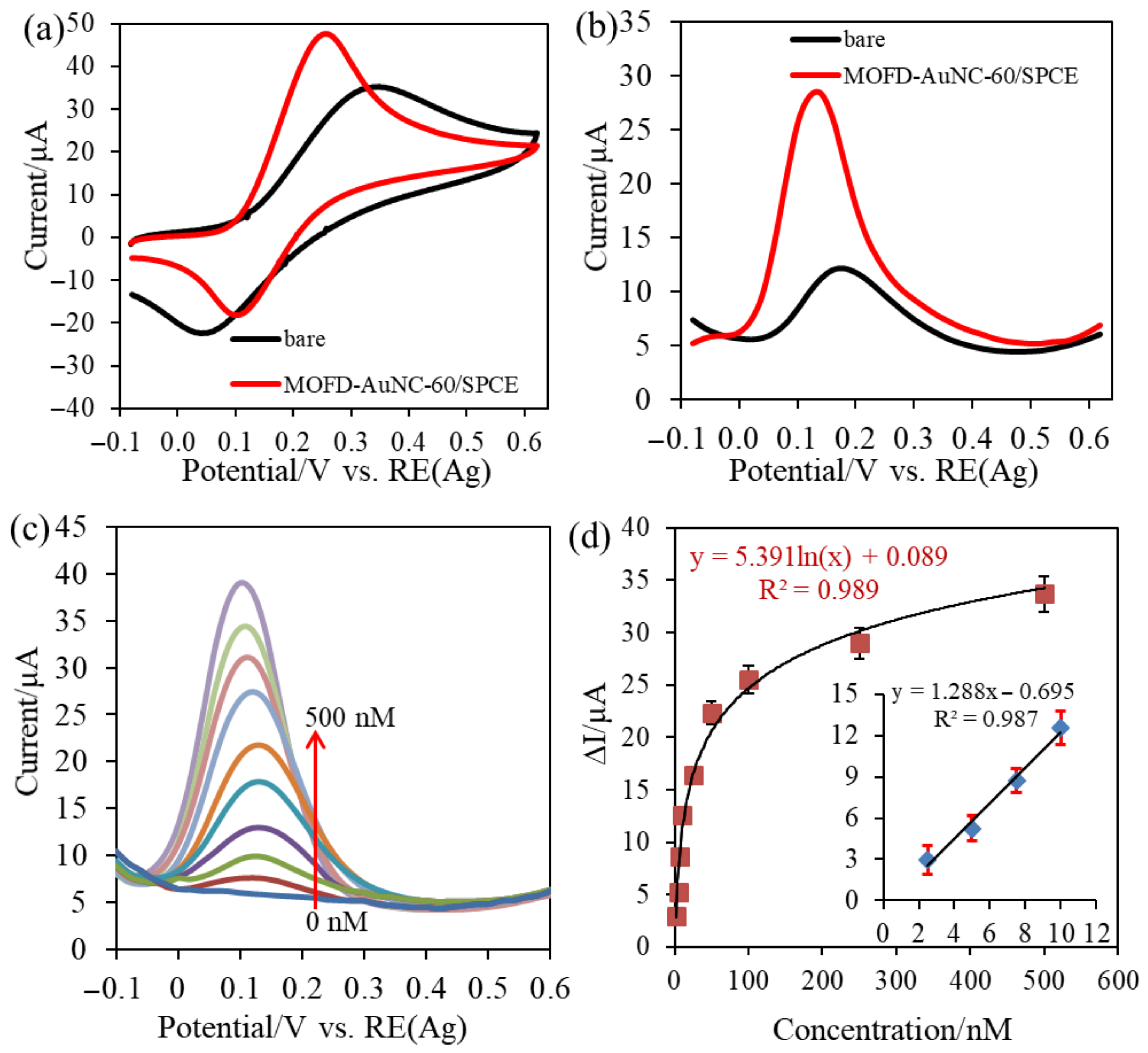
(**a**) CV and (**b**) DPV responses of dopamine (100 nM) measured at bare and MOFD-AuNC-60-modified SPCEs. (**c**) The DPV measurement of DA (0, 2.5, 5, 7.5, 10, 25, 50, 100, 250, 500 nM)-containing PBS and (**d**) the corresponding calibration curve obtained from three sensors. The inset shows the current responses to 2.5−10 nM DA.

Figure 3a,b show the DA results (100 nM) measured by CV and DPV at the bare SPCEs and MOFD-AuNC-60/SPCEs, respectively. Obviously, the MOFD-AuNC-60 coating showed lower E_pa_ and larger I_pa_ than the bare SPCEs, suggesting a faster electron-transfer rate and a larger area. Moreover, the potential difference in E_pa_–E_pa/2_, where E_pa/2_ is the half-peak potential measured at the SPCE and the MOFD-AuNC-60/SPCE was 0.159 V and 0.099 V, respectively. In principle, the E_pa_–E_pa/2_ value is a peak shape function (Λ = *k*°(RT/DF*v*)^0.5^ of a quasi-reversible reaction, where *k*^o^ is the heterogeneous electron-transfer rate constant, R is the gas constant, T is the absolute temperature, D is the diffusion coefficient of DA, F is the Faraday constant, and *v* is the scanning rate [36]. The smaller E_pa_–E_pa/2_ indicates that the MOFD-AuNC-60 coating can promote the DA’s *k*° value to act as a nanozyme on the SPCEs. Similarly, in Figure 3b, the lower E_pa_ and the larger I_pa_ measured by DPV at MOFD-AuNC-60/SPCEs than at bare SPCEs indicate that the MOFD-AuNC-60 coating promotes faster electron transfer and greater roughness.

Furthermore, Figure S4a,b (Supplementary Information) show the CV results of low (5 nM) and high (250 nM) DA concentrations with different *v* to explore the reaction mechanism of DA on the MOFD-AuNC-60/SPCEs. Figure S4c,d show the fitting curves of I_pa_ versus *v* and *v*^0.5^. The coefficient of determination (*R*^2^) between I_pa_ and *v* for 5 nM DA, being 0.977, is larger than the that (0.954) between I_pa_ and *v*^0.5^, implying that the electrochemical mechanism of low concentration DA on the MOFD-AuNC-60/SPCEs presents a higher tendency to surface adsorption [37], attributed to the electric interaction between the negatively charged BTC and the positively charged DA. At a high concentration (250 nM), the *R*^2^ values for *v* and *v*^0.5^ differ little, suggesting that diffusion and adsorption simultaneously dominate the electrochemical reaction.

Figure 3c shows the DPV responses in PBS containing DA at concentrations of 2.5–500 nM. For analytical evaluation, ΔI was defined as the current difference measured at the E_pa_ position between the DA-containing solutions and the blank solution. As shown in Figure 3d, the responses over 2.5–500 nM were well fit by a logarithmic relationship, y = 5.391 ln(x) + 0.089, with a coefficient of determination (R^2^) of 0.989. The LOD was estimated at 2.7 nM based on S/N = 3, using the calibration slope from the low-concentration linear region (2.5–10 nM), with the regression equation ΔI (μA) = 1.288 C_DA_ (nM) − 0.695. Accordingly, the practical dynamic range was defined as 5–500 nM, from the lowest tested concentration above the LOD to the highest tested concentration.

### 3.5. Selectivity and Sensing Properties of DA in the Presence of AA and UA

Figure S5 shows the DPV of the DA (100 nM)/AA (100 nM)/UA (100 nM) mixture. The corresponding E_pa_ values for DA, AA, and UA were 0.05 V, 0.24 V, and 0.40 V, respectively. The significant E_pa_ separation between them indicates good selectivity for distinguishing the oxidation signals of DA, AA, and UA. Moreover, the I_pa_ of DA oxidation was higher than that of AA and UA, suggesting that the MOFD-AuNC-60 coating has a faster electron-transfer rate for DA than for AA and UA. Notably, the DA E_pa_ (0.05 V) detected by DPV in this study is lower than those (0.11–0.3 V) of previous nanomaterial-based studies [12,13,15,16,19,20,21,24]. Moreover, the MOFD-AuNC-60/SPCEs exhibit DA’s E_pa_ earlier than that of AA and UA, demonstrating excellent selectivity for DA detection with minimal interference from AA and UA.

**Figure 4:**
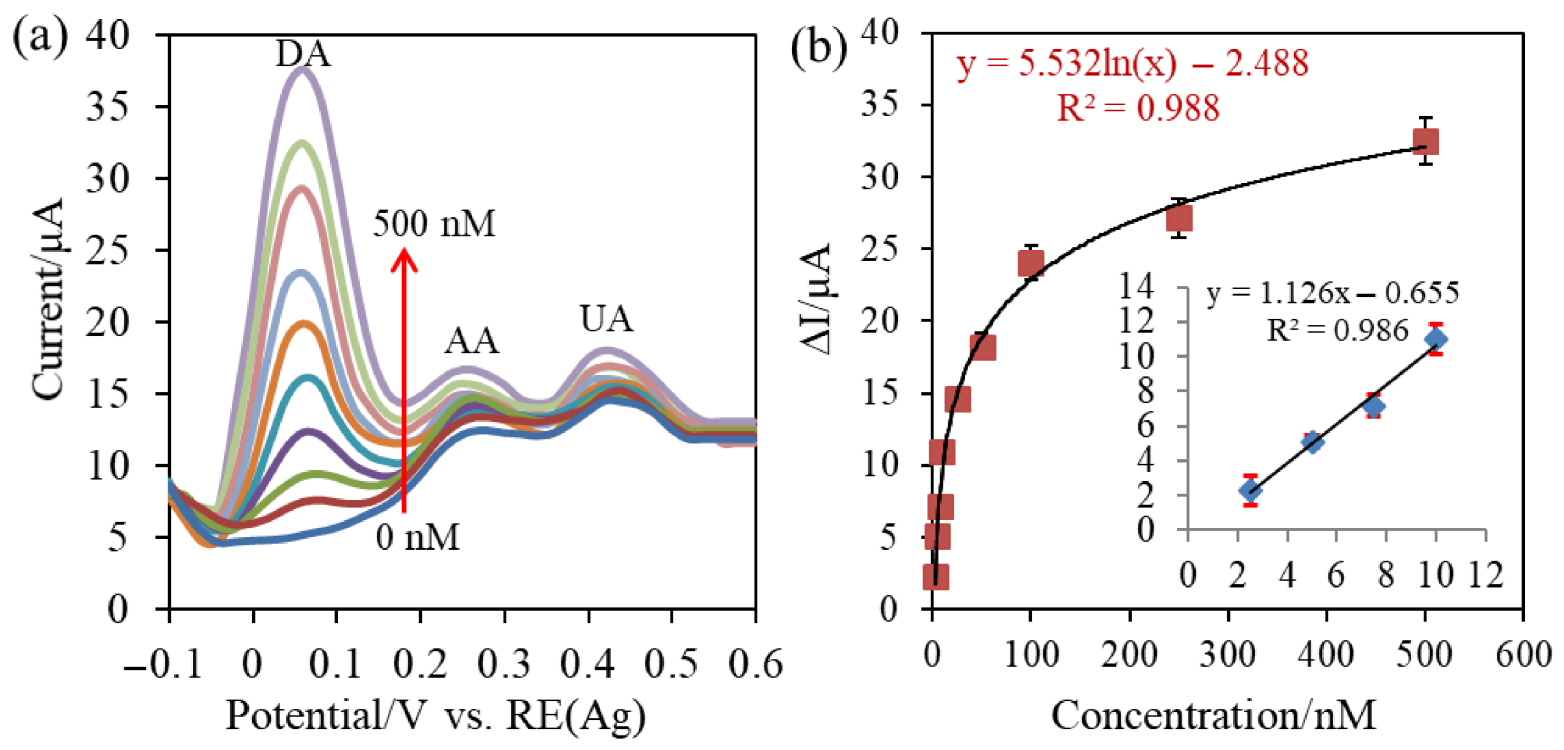
(**a**) DPV measurement of the PBS-based mixtures, including concentration-varied DA (0, 2.5, 5, 7.5, 10, 25, 50, 100, 250, 500 nM), AA (100 nM), and UA (100 nM) obtained at the MOFD-AuNC-60/SPCEs and (**b**) the corresponding calibration curve obtained from three individual sensors. The inset shows the current responses to 2.5−10 nM DA.

The voltammograms and corresponding calibration curves for DA detection in the presence of 100 nM AA and 100 nM UA are shown in Figure 4. As shown in Figure 4a, the ΔI value corresponding to DA increased with increasing DA concentration. In contrast, the responses attributed to AA and UA remained nearly unchanged, indicating good electrochemical selectivity toward DA. As shown in Figure 4b, the LOD was estimated to be 2.8 nM from the calibration slope in the low-concentration region (2.5–10 nM), following the regression equation ΔI (μA) = 1.126 C_DA_ (nM) − 0.655 (R^2^ = 0.986). Moreover, the sensor exhibited a dynamic range of 5–500 nM. These results indicate that the sensor retained a reliable concentration-dependent response to DA even in the presence of AA and UA. Furthermore, within the low-concentration region of 2.5–10 nM, the calibration slope obtained in the DA/AA/UA mixture (Figure 4b) remained 87.4% of that in the pure DA solution (Figure 3d). This minor decrease indicates that the presence of AA and UA caused a slight variation in sensitivity and did not adversely affect DA detection.

### 3.6. DA Detection in Serum Samples

**Figure 5:**
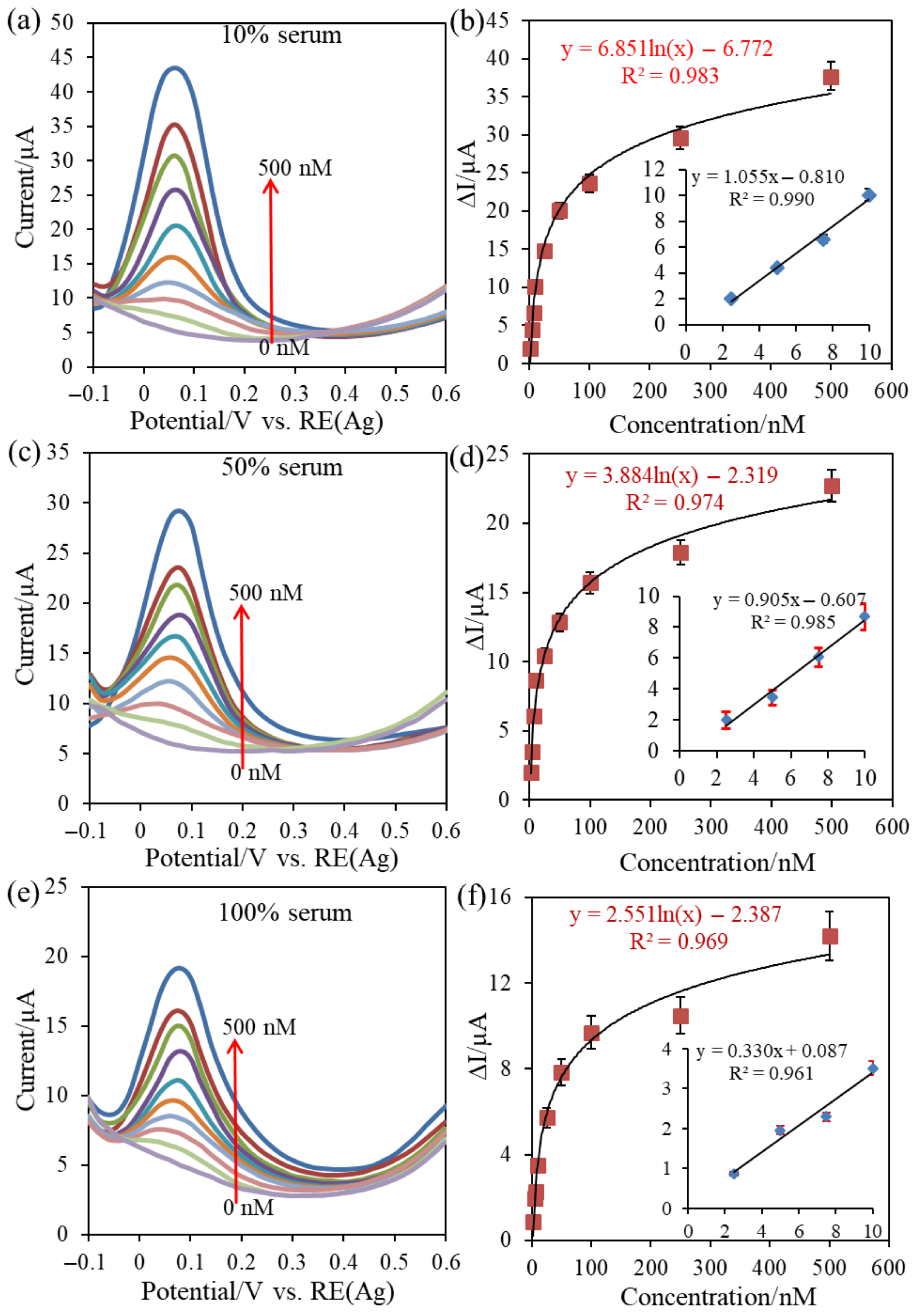
DPV curves of concentration-varied DA (0, 2.5, 5, 7.5, 10, 25, 50, 100, 250, 500 nM) spiked in (**a**) 10%, (**c**) 50%, and (**e**) 100% porcine serum. The corresponding calibration curves are shown in (**b**,**d**,**f**), with insets showing the linear range of 2.5–10 nM. The statistical data are calculated from at least three individual sensors.

Serum samples contain various electroactive species and proteins that may interfere with analyte detection. Therefore, DA at concentrations ranging from 2.5 to 500 nM was spiked into 10% and 50% porcine serum diluted with PBS, as well as undiluted porcine serum. The corresponding DPV responses are shown in Figure 5a,c,e. In all serum samples, the oxidation peak potential (E_pa_) of DA was observed at approximately 0.05 V versus the integrated Ag-RE of the SPCE, suggesting that the serum matrix had little effect on the DA oxidation potential. The corresponding ΔI values are summarized in Figure 5b,d,f. For analytical evaluation, the low-concentration range of 2.5–10 nM was used to estimate the LOD values. The regression equations obtained from this linear region were ΔI (μA) = 1.055 C_DA_ (nM) − 0.810 for 10% serum, ΔI (μA) = 0.905 C_DA_ (nM) − 0.607 for 50% serum, and ΔI (μA) = 0.330 C_DA_ (nM) + 0.087 for undiluted serum. The corresponding LOD values were estimated to be 2.5, 2.6, and 0.5 nM, respectively. Based on the lowest tested concentrations above the corresponding LOD values, the dynamic ranges were defined as 2.5–500 nM for 10% serum, 5–500 nM for 50% serum, and 2.5–500 nM for undiluted serum. These results demonstrate the potential of the MOFD-AuNC-60/SPCE strips for practical DA detection in complex biological samples. Moreover, the relative standard deviation (RSD) values calculated from the ΔI responses were all below 4%, indicating good reproducibility. Furthermore, the calibration slope decreased as serum concentration increased. This trend may be attributed to the higher viscosity and greater biomolecular content of more concentrated serum, which could reduce the diffusion coefficient of DA and hinder its access to the electrode surface. Table S2 shows the recovery values for 10 nM, calculated from the calibration curves in 10%, 50%, and 100% porcine serum, which range from 102.5% to 103.4%. The results elucidate that the sensors have good recovery.

The stability of the DA sensors is crucial for their practical application. Figure S6 shows three individual MOFD-AuNC-60/SPCEs for detecting 10 nM DA spiked in 10% porcine serum. The ΔI value of the 7-day measurement slightly decreased from 10.11 ± 0.30 A (1st day) to 9.35 ± 0.32 A (7th day), a 7.5% decrease, indicating good stability. Furthermore, the RSD calculated from all measurements was less than 3%, indicating good reproducibility.

**Figure 6:**
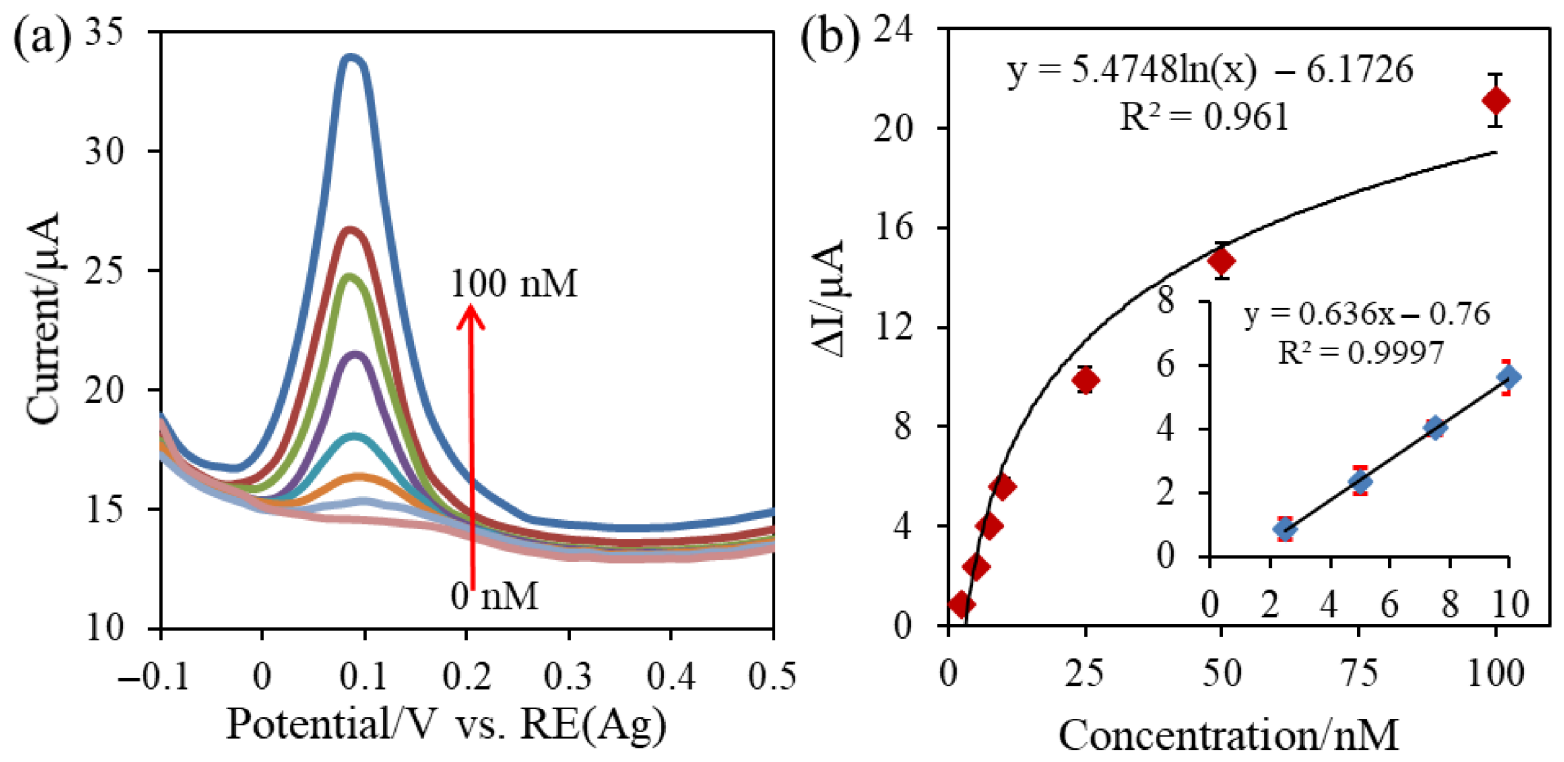
(**a**) DPV curves of concentration-varied DA (0, 2.5, 5, 7.5, 10, 25, 50, 100 nM) spiked in undiluted human serum. (**b**) The corresponding calibration curves are shown in (**b**), with insets showing the linear range from 2.5 to 10 nM. The statistical data are calculated from at least three individual sensors.

Figure 6 presents the DPV responses and corresponding calibration curves for DA detection in undiluted commercial human serum using the MOFD-AuNC-60/SPCEs over a tested concentration range of 2.5–100 nM. For analytical evaluation, the low-concentration region of 2.5–10 nM was used to estimate the LOD, following the regression equation ΔI (μA) = 0.636 C_DA_ (nM) − 0.760. The LOD was estimated to be 4.4 nM. Based on the lowest tested concentration above the LOD, the dynamic range was defined as 5–100 nM. The calibration slope in the low-concentration range of 2.5–10 nM was higher than in undiluted porcine serum but lower than in PBS. This difference may be attributed to variations in solution viscosity and molecular composition among the tested media. Table S3 shows the recovery ratios for 2.5, 5, 7.5, and 10 nM, ranging from 98.4% to 102.0%. The results elucidate that the sensors have good recovery in undiluted serum samples. Furthermore, the DPV baseline curves in Figure 3c, Figure 5e and Figure 6a differed among the tested solution systems, mainly because the background current outside the DA oxidation region was governed by the double-layer capacitive current at the electrode/electrolyte interface, which is sensitive to the electrode surface condition and the solution composition.

Table 1 summarizes the sensing performance of the MOFD-AuNC-60/SPCEs and previously NC-based DA sensors [12,13,15,16,19,20,21,22,24,38]. The MOFD-AuNC-60/SPCEs achieved the relatively low LOD among these DA sensors. This comparison suggests that the MOFD-AuNC-60 coating effectively enhances the electrochemical detection performance of DA on SPCEs. Notably, unlike earlier studies that relied on highly diluted biological matrices (≤1% serum or urine), our sensor enables direct detection of DA in undiluted serum. Furthermore, the dynamic range of 5–100 nM covers clinically relevant DA concentrations [3,4,5] without requiring sample dilution, thereby enabling rapid analysis.

## 4. Conclusions

In summary, MOFD-AuNCs were successfully synthesized, and a single coating of MOFD-AuNC-60 on SPCEs markedly improved the electrochemical performance toward DA detection. The MOFD-AuNC-60/SPCEs exhibited high selectivity for DA electrooxidation, with a lower oxidation overpotential than that of AA and UA. DPV measurements demonstrated a dynamic range of 5–100 nM and an LOD of 4.4 nM in undiluted human serum, indicating competitive analytical performance compared with previously reported studies. Moreover, the proposed sensor enabled direct detection of DA in undiluted serum samples, highlighting its potential for clinically relevant DA analysis. Together with its good reproducibility and stability, these results demonstrate that the MOFD-AuNC-60-coated SPCEs provide a robust and reliable electrochemical sensing platform for practical applications.

## Figures and Tables

**Table 1:** Comparison of the electrochemical sensing properties between the MOFD-AuNC-60/SPCEs and previous NC-based DA sensors.

NC-Modified Electrodes	Dynamic Range (μM)	LOD (nM)	Real Samples/Epa of DA by DPV	Refs.
CNC-CuTMePP-MOF/GCE	0.1–100	64	Human serum with a 43-fold dilution by PBS/0.21 V	[12]
N-TiO_2_@AgNPs/GQD/GCE	0.003–300	1	1 nM DA in human serum with 2% methanol, centrifugation, and a 10-fold dilution by PBS/0.11 V	[13]
AuNPs-MWCNT/GO/GCE	1–150	280	2.1 μM DA in diluted plasma by PBS/0.25 V Epa	[15]
GO-Au@Cu-MOF/SPCE	0.1–100	16	50 μM DA in human serum with a 500-fold dilution by PBS/0.1 V	[16]
ZIF-8/SPCEs	2–50	87	Only PBS/0.21 V	[19]
Cu_3_(HHTP)_2_-MOF /GCE	0.05–200	5.1	0.1 μM DA in human serum/0.3 V	[20]
Co/Zn-TCPP-MOF/GCE	0.005–178	1.7	Only PBS/0.1 V	[21]
Co-N, S-C/GCE	5–115	430	1 μM DA in a 100-fold dilution of human urine by SWV	[22]
L-Co-MOF/CC	0.0075–50	2.5	5 μM DA in human serum with a 100-fold dilution by PBS/0.22 V	[24]
Au@Ni-MOF/SPCE	0.5–1000	140	5 μM DA in human serum with a 200-fold dilution by PBS/0.2 V	[38]
MOFD-AuNC-60/SPCE	2.5–500 nM 5–100 nM	0.5 4.4	Undiluted porcine serum/0.08 V Undiluted human serum/0.08 V	[This Work]

## Data Availability

Data will be made available on request.

## Supplementary Materials

The following supporting information can be downloaded at https://www.mdpi.com/article/10.3390/bios16050255/s1, Figure S1: TEM images of Au^3+^-BTC MOF (a) and MOFD-AuNC-60 (b). The inset shows octahedral structures; Figure S2: FTIR spectrum of Au^3+^-BTC NCs (black) and MOFD-AuNC-60 (red); Figure S3: SEM images of bare oxidized SPCEs (a) and the MOFD-AuNC-60/SPCEs; Figure S4: Cyclic voltammograms of MOFD-AuNC-60/SPCEs measured in 5 nM (a) and 250 nM (b) DA-containing PBS with scanning rates ranging from 10 to 300 mV/s. (c) and (d) are the corresponding relation curves between the anodic peak current versus the scanning rate (c) and the square root of the scanning rate (d). The statistical data is obtained from three individual sensors; Figure S5: DPV curve of the PBS-based mixture of 100 nM DA, 100 nM AA, and 100 nM UA measured at MOFD-AuNC-60/SPCEs; Figure S6: Stability test of MOFD-AuNC-60/SPCEs sensors for 10 nM DA spiked in 10% porcine serum (*n* = 3); Table S1: The XRD analysis of MOFD-AuNC-30, MOFD-AuNC-60, and MOFD-AuNC-120, including peak position (2θ), Miller indices (hkl), and crystallinity percentage (%), is compared with the characteristic faces of MOF, BTC, and gold nanoparticles; Table S2: The DA concentration found in concentration-varied porcine serum samples was calculated from the calibration curves of Figure 5b,d,f (*n* = 3); Table S3: Detection of DA in undiluted human serum samples (*n* = 3).

## Abbreviations

The following abbreviations are used in this manuscript:

AA	Ascorbic acid
BTC	1,3,5-Benzene tricarboxylic acid
CV	Cyclic voltammetry
DA	Dopamine
DPV	Differential pulse voltammetry
EIS	Electrochemical impedance spectroscopy
GCE	Glassy carbon electrode
LOD	Limit of detection
MOFD-AuNC	Metal–organic framework-derived gold nanocomposite
PBS	Phosphate-buffered solution
RSD	Relative standard deviation
SPCE	Screen-printed carbon electrodes
SWV	Square wave voltammetry
UA	Uric acid
