# Supplementary material for: Ultrasensitive Dopamine Detection in Undiluted Serum with a Disposable Electrochemical Sensor Employing MOF-Derived Gold Nanocomposites
Source: Biosensors (Basel). 2026 Apr 30;16(5):255. doi: 10.3390/bios16050255 (PMC13204897; doi:10.3390/bios16050255)
Supplement: Supplementary file 1 [file biosensors-16-00255-s001.zip › biosensors-4223322-supplementary.pdf]

Supplementary material

Ultrasensitive Dopamine Detection in Undiluted Serum with a Disposable Electrochemical Sensor Employing MOF-Derived Gold Nanocomposites

Rohan Sagar[1], Hsiao-Wei Wen[2], Ching-Chou Wu[1,3*] and M. S. Gaur[4]

[1]Department of Bio-industrial Mechatronics Engineering, National Chung Hsing University, Taichung City, Taiwan, ROC
[2]Department of Food Science and Biotechnology, National Chung Hsing University, Taichung 402, Taiwan, ROC
[3]Development Center of Sustainable Agriculture, National Chung Hsing University, Taichung City, Taiwan, ROC
[4]Department of Physics, Hindustan College of Science and Technology, Farah, Mathura, Uttar Pradesh 281122, India, affiliated to Dr. A.P.J. Abdul Kalam Technical University, Lucknow, Uttar Pradesh 226031, India
*Corresponding author: Ching-Chou Wu **E-mail**: ccwu@dragon.nchu.edu.tw

List of Figures

S1. TEM images of Au^{3+}-BTC NCs (a) and MOFD-AuNC-60 (b). The inset shows octahedral structures.
S2. FTIR spectrum of Au^{3+}-BTC NCs (black) and MOFD-AuNC-60 (red)
S3. SEM images of bare oxidized SPCEs (a) and the MOFD-AuNC-60/SPCEs
S4. Cyclic voltammograms of MOFD-AuNC-60/SPCEs measured in 5 nM (a) and 250 nM (b) DA-containing PBS with scanning rates ranging from 10 to 300 mV/s. (c) and (d) are the corresponding relation curves between the anodic peak current versus the scanning rate (c) and the square root of the scanning rate (d). The statistical data is obtained from three individual sensors
S5. DPV curve of the PBS-based mixture of 100 nM DA, 100 nM AA, and 100 nM UA measured at MOFD-AuNC-60/SPCEs
S6. Stability test of MOFD-AuNC-60/SPCEs sensors for 10 nM DA spiked in 10% porcine serum (n=3)

List of Tables

S1. The XRD analysis of MOFD-AuNC-30, MOFD-AuNC-60, and MOFD-AuNC-120, including peak position (2θ), Miller indices (hkl), and crystallinity percentage (%), is compared with the characteristic faces of MOF, BTC, and gold nanoparticles.
S2. The DA concentration found in concentration-varied porcine serum samples was calculated from the calibration curves of Figures 5(b , d, f) (n=3).
S3. Detection of DA in undiluted human serum samples (n=3).

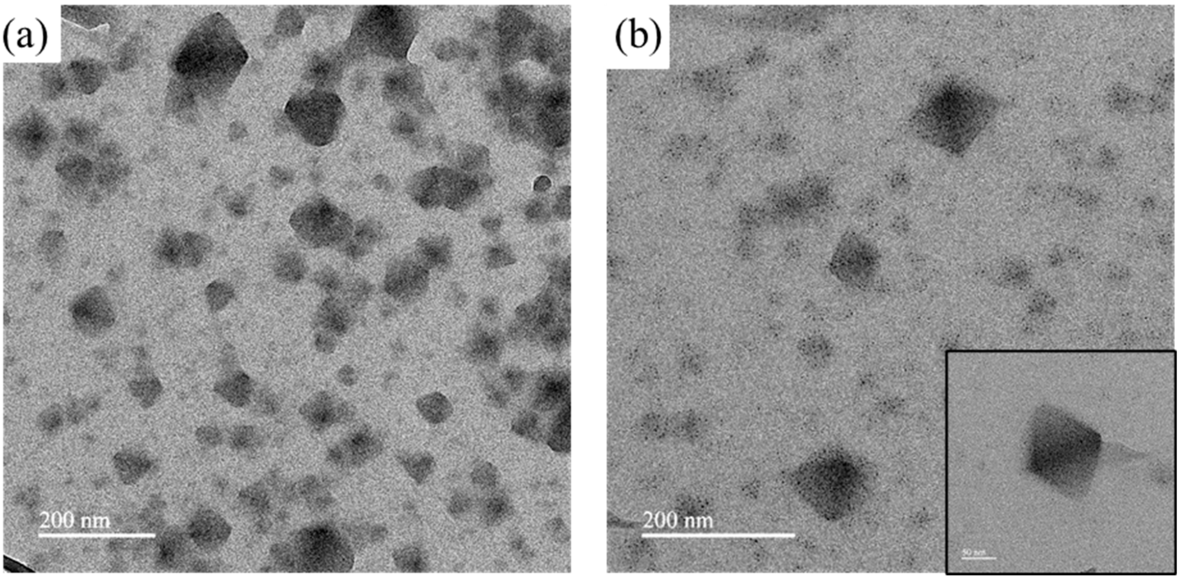

Figure S1. TEM images of Au^{3+}-BTC NCs (a) and MOFD-AuNC-60 (b). The inset shows octahedral structures

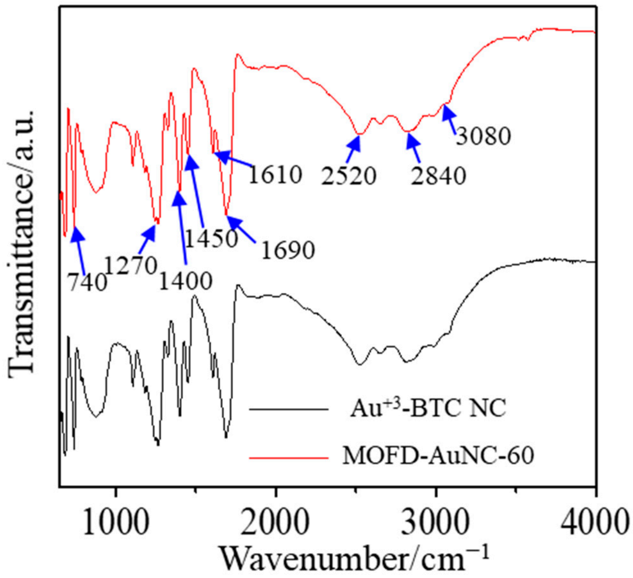

Figure S2. FTIR spectrum of Au^{3+}-BTC NCs (black) and MOFD-AuNC-60 (red).

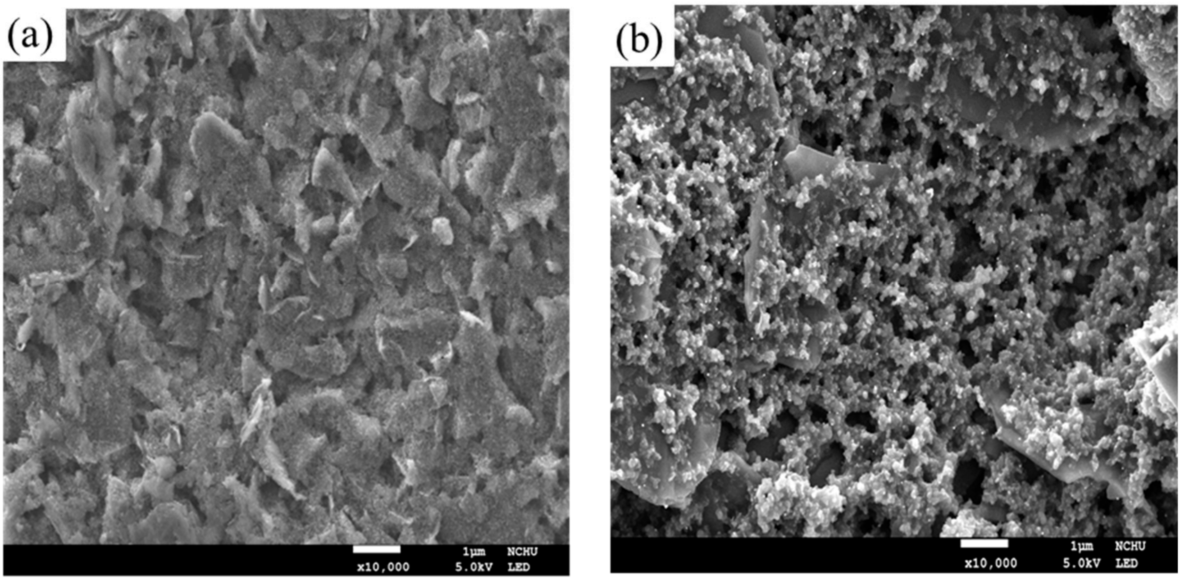

Figure S3. SEM images of bare oxidized SPCEs (a) and the MOFD-AuNC-60/SPCEs

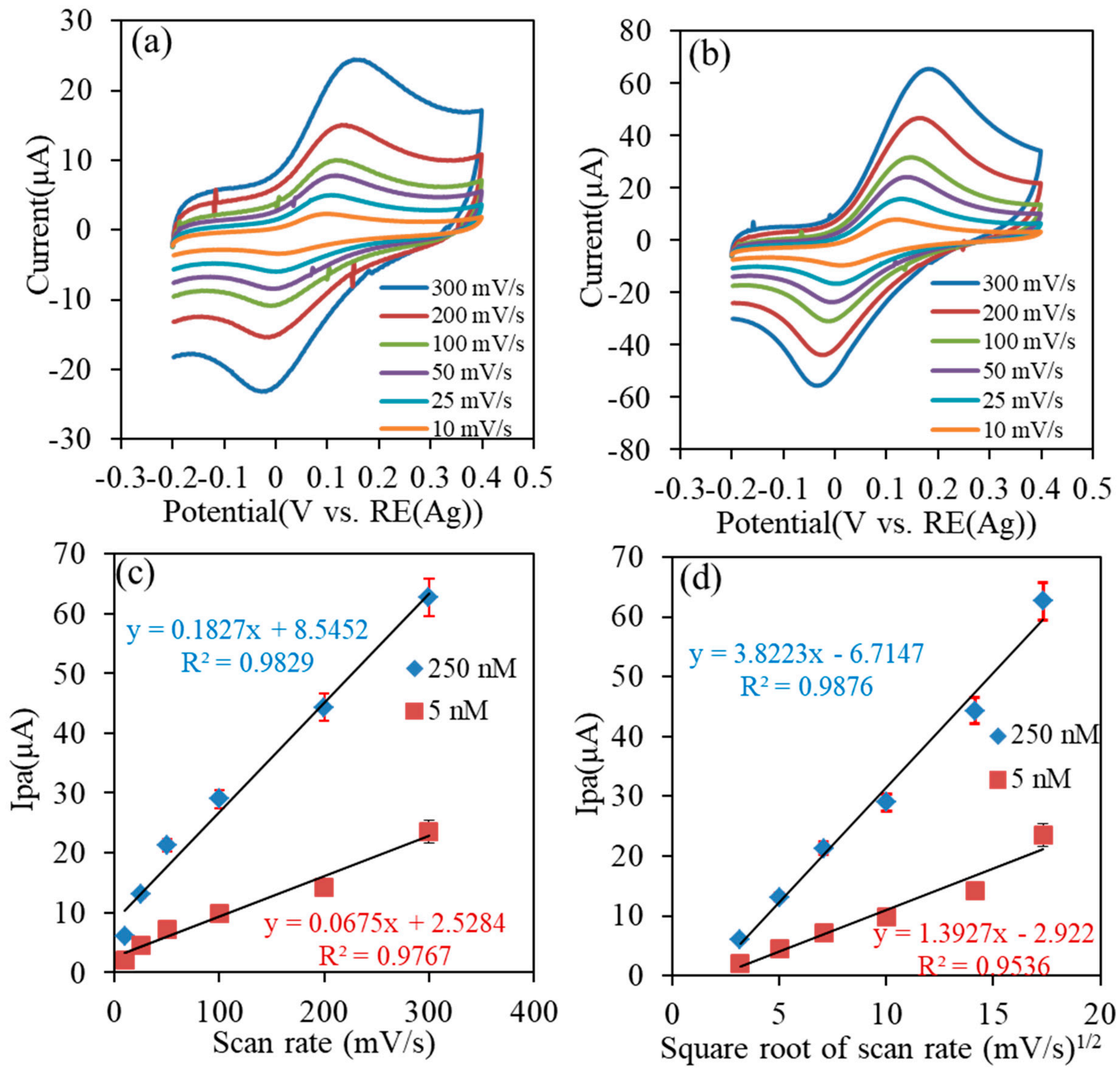

Figure S4. Cyclic voltammograms of MOFD-AuNC-60/SPCEs measured in 5 nM (a) and 250 nM (b) DA-containing PBS with scanning rates ranging from 10 to 300 mV/s. (c) and (d) are the corresponding relation curves between the anodic peak current versus the scanning rate (c) and the square root of the scanning rate (d). The statistical data is obtained from three individual sensors

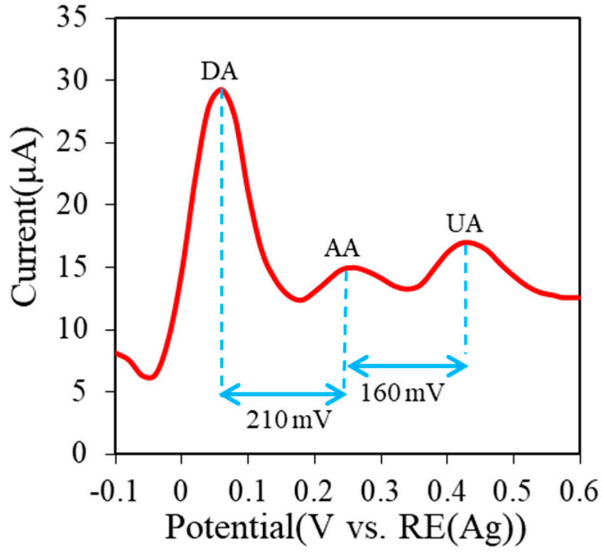

Figure S5. DPV curve of the PBS-based mixture of 100 nM DA, 100 nM AA, and 100 nM UA measured at MOFD-AuNC-60/SPCEs

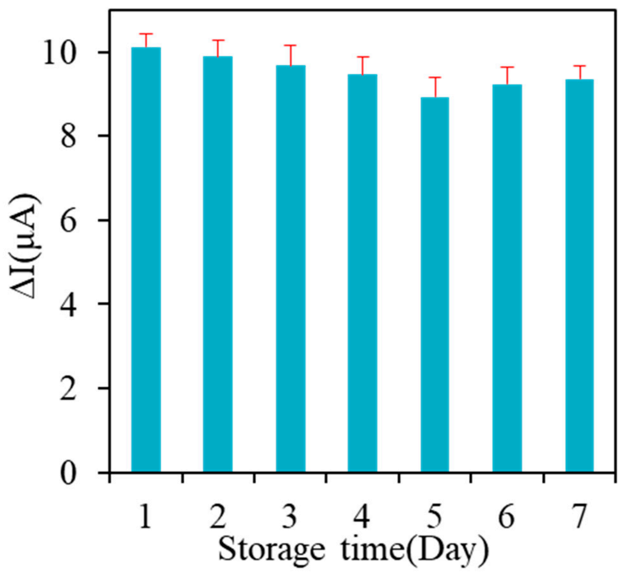

Figure S6. Stability test of MOFD-AuNC-60/SPCEs sensors for 10 nM DA spiked in 10% porcine serum (n=3)

Table S1. The XRD analysis of MOFD-AuNC-30, MOFD-AuNC-60, and MOFD-AuNC-120, including peak position (2θ), Miller indices (hkl), and crystallinity percentage (%), is compared with the characteristic faces of MOF, BTC, and gold nanoparticles

**2θ (°)**	**Crystallinity percentage (%)[1]**			**Material features**		
	**MOFD-AuNC-30**	**MOFD-AuNC -60**	**MOFD-AuNC-120**	**BTC**	**AuNPs**	**MOF**
6.5–6.7	65	71	70	(010)-Layered BTC structure	No significant peak	(010)-MOF periodicity
10.6–10.8	70	86	82	(100) – BTC stacking	No significant peak	(220)-MOF reflection
18.2–18.3	83	87	85	(210) -BTC structure, hydrogen bonding	No significant peak	(210)-MOD periodicity
24.2–24.6	62	80	78	(300) – Long-range order	No significant peak	(300)-MOF reflection
27.5–27.7	57	73	68	(311)-Hydrogen bonding	No significant peak	(311)-MOF overlap
29.4–29.7	54	64	60	(110) – BTC benzene stacking	No significant peak	(110)-MOF-metal interaction
38.5–38.8	43	49	48	No peak	(111), (200)-Au FCC faces	(111)-MOF diffraction

[1]Crystallinity (%) from X-ray diffraction (XRD) data is commonly calculated using the peak area method, where the ratio of the area under crystalline peaks to the total area (crystalline + amorphous) is determined. This involves deconvoluting the XRD pattern into sharp crystalline peaks and broad amorphous regions using software like Origin or X'Pert High Score. The crystallinity is then calculated using the formula: Crystallinity (%) = $(A_c / (A_c + A_a)) \times 100$, where A_c is the area under crystalline peaks and A_a is the area under the amorphous hump. This method provides a quantitative estimate of the crystalline structure within a sample.

Table S2. The DA concentration found in concentration-varied porcine serum samples was calculated from the calibration curves of Figures 5(b, d, f) (*n*=3)

Serum	Added (nM)	Found (nM)	Recovery (%)
10%	10	10.31	103.1
50%	10	10.25	102.5
100%	10	10.34	103.4

Table S3. Detection of DA in undiluted human serum samples (n=3)

Added (nM)	Found (nM)	Recovery (%)
2.5	2.55	102.0
5.0	4.92	98.4
7.5	7.53	100.4
10.0	10.00	100.0
